# Development and Initial Psychometric Testing of a Patient-Reported Clinical Tool for Endometriosis: The Mobility Measure for Endometriosis (MobEndo)

**DOI:** 10.3390/jcm15072765

**Published:** 2026-04-06

**Authors:** Joaquina Montilla-Herrador, Mariano Gacto-Sánchez, Jose Lozano-Meca, Mariano Martínez-González, María Pilar Marín Sánchez, Francesc Medina-Mirapeix

**Affiliations:** 1 Faculty of Medicine, CEIR Campus Mare Nostrum CMN, University of Murcia and Biomedical Research Institute of Murcia Pascual Parrilla-IMIB, 30120 Murcia, Spain; montilla@um.es (J.M.-H.); mamargon@um.es (M.M.-G.); mirapeix@um.es (F.M.-M.); 2 Faculty of Nursing and Physiotherapy, University of the Balearic Islands, 07122 Palma de Mallorca, Spain; jose.lozano@uib.es; 3 Department of Gynaecology and Obstetrics, Hospital General Universitario Santa Lucía, University of Murcia and Biomedical Research Institute of Murcia Pascual Parrilla-IMIB, 30120 Murcia, Spain; mp.marin@carm.es

**Keywords:** mobility, endometriosis, questionnaire, patient-centered, disability

## Abstract

**Background:** Women with endometriosis frequently experience mobility limitations that affect daily functioning. A specific tool to assess these restrictions would help clinicians to better understand patients’ functional challenges, facilitating more effective communication and shared decision making. Addressing this gap is essential for strengthening patient–professional dialogue and improving individualized care. **Objective:** To develop the new instrument MobEndo and to perform initial psychometric testing of the tool. **Methods:** The initial domains and items were generated through semi-structured interviews with patients and based on experts’ advice. Guided by the International Classification of Functioning, Disability, and Health (ICF) framework, exploratory factor analysis was conducted on data from patients diagnosed with endometriosis. Internal consistency was assessed using Cronbach’s alpha, considering values ≥ 0.70 as acceptable. Test–retest reliability was examined using intraclass correlation coefficients (ICCs), and ICC values were judged as excellent if >0.75. Construct validity was evaluated through concurrent, discriminant, and known-groups validity. For the known-groups validity hypothesis, participants were categorized by baseline pain levels. **Results:** The final questionnaire included 18 items, developed from responses from 301 women (mean age 38.96 ± 6.85). Factor analysis revealed two components—transitioning between body positions and performing movements requiring stabilization and executing load-bearing tasks involving the upper limbs—with the model explaining 71.78% of the total variance. Reliability was excellent, with a Cronbach’s alpha of 0.977. The ICC for the total score was 0.976 (95% CI 0.949–0.988), with similarly high values for each component. Concurrent validity correlations were significant, while discriminant validity showed no relevant associations. Known-groups analyses showed clear differences across pain-level groups. **Conclusions:** The questionnaire is a valid and reliable tool for capturing women’s perceived mobility limitations in endometriosis.

## 1. Introduction

Endometriosis is a chronic gynecological condition characterized by the presence of endometrial-like tissue outside the uterus. It affects up to 10% of women worldwide, and the exact age of onset is difficult to estimate, since the diagnosis is often delayed in a 5-to-12 year range after the real onset of symptoms [1]. Endometriosis often leads to pelvic chronic pain, dysmenorrhea, dyspareunia, and other symptoms that significantly impair daily life [1,2]. Compared to women without endometriosis, those with the condition experience significantly greater menstrual pain severity, more intense symptoms and fatigue, widespread pain sites, marked functional disability related to pain, and poorer health-related quality of life [3].

Many women experiencing chronic pain report moderate to severe limitations to mobility [2]. Multiple studies have consistently demonstrated that pain symptoms substantially impair health-related quality of life, particularly in the physical domain, therefore underscoring the strong association between physical functioning and pain in this population [4,5,6]. Despite its high prevalence and profound impact on quality of life, in the field of endometriosis, no specific tools to measure women’s limitations in these activities currently exist. Most of the existing tools and endometriosis questionnaires testing mobility activities only focus on the impact of pain on the aforementioned activities as a proxy for measuring health-related quality of life. For example, the pain scale of the Endometriosis Health Profile (EHP) questionnaire [7] measures whether frequent pain impacts three mobility activities (i.e., standing, sitting, and walking). The Endometriosis Impact Questionnaire (EIQ) [8] also measures, indirectly, whether pain due to pelvic pain or menstruation impacts these activities over long periods of time, by asking about the time spent time lying down.

Since the existing assessment tools mainly focus on pain intensity, domains such as physical function remain somewhat underexplored, representing a gap in the comprehensive evaluation of functional limitations amongst women with endometriosis [9]. On another note, even though previous research has documented some mobility limitations, no previous study has focused on systematic measurement grounded on the International Classification of Functioning, Disability, and Health (ICF) [10] to assess whether limitations differ across types of mobility activities (e.g., changing and maintaining body position; carrying, moving, and handling objects; walking and moving) and whether these limitations are accompanied by pain, other sensations (effort, slowness), or both aspects concurrently. Furthermore, while some studies have shown that women with endometriosis display decreased capacity or intrinsic ability in standardized performance-based mobility tests, such as walking and chair-stand tests in clinical settings [11,12], the gap between functional capacity and the perception of performance in real environments remains unexplored, and thus unknown.

There is growing interest in integrating both structured questions and patient-reported outcome measures (PROMs) in consultations with women affected by endometriosis, in order to better understand the complexity of their symptoms and the overall disease burden [13,14,15]. The conception of a specific PROM would lead to a better understanding of the global picture of functional limitations amongst women with endometriosis, therefore improving and enhancing active patient involvement and communication and, ultimately, patient care, three aspects that have been identified as cornerstones of future improvements in the care of patients with endometriosis [16]. Creating a novel instrument based on patient-reported scores would also enable accurate goal setting and prioritization of patient needs and would also improve the design of tailored interventions, thereby improving patient–clinician relationships [17], which, in turn, is a powerful driver to ultimately improve healthcare outcomes [18]. This strong need to foster and support a positive relationship between patients and care providers has already been explored and highlighted across different conditions [18], and it is even more pronounced in women with endometriosis [19].

In order to contribute to the development of further knowledge on the aforesaid matters, this study aimed to develop and analyze the psychometric properties of a new patient-reported instrument called MobEndo for capturing the perceptions of women with endometriosis regarding their mobility limitations. More specifically, we hypothesized that the factor structure of the instrument would capture the three following domains of mobility activities of the ICF framework: changing and maintaining body position; carrying, moving, and handling objects; and walking and moving.

## 2. Materials and Methods

### 2.1. Study Sample

A sample of 301 women with a confirmed medical diagnosis of endometriosis was recruited from a hospital setting. The diagnosis of endometriosis complied with the guidelines of the European Society of Human Reproduction and Embryology (ESHRE), which means that the diagnosis was derived from visual detection of endometriotic lesions during previous surgeries, anatomopathological tests, and typical ultrasonographic features of endometriosis [20]. Patients with suspected endometriosis but without surgical or ultrasound confirmation were excluded. No specific information on the potential stage of the disease was collected. Patients were eligible if they were 18 to 50 years old and if they were able to read, understand, and speak Spanish fluently. Patients were excluded if they had any condition that could compromise data quality (e.g., severe auditory, intellectual, or visual impairments, comorbidities, or menstruation preventing proper completion of questionnaires or physical tests) or they had received specific analgesic interventions (e.g., infiltrations, nerve blocks, or morphine pumps) within the 4 weeks previous to the study onset.

### 2.2. Measures

The initial domains and items for MobEndo were identified through semi-structured interviews with patients and based on experts’ advice. Guided by the three domains of mobility activities of the ICF framework (changing and maintaining body position; carrying, moving, and handling objects; walking and moving) [10], we aimed to pinpoint specific actions, postures, and movements commonly performed with difficulty or limitation (and thus posing challenges) in daily life for individuals with endometriosis. The interviewers carefully observed participants’ comments and nonverbal cues (gestures) to facilitate accurate reporting of limitations in mobility activities. We adopted the framework of the Manual for WHO Disability Assessment Schedule (WHODAS 2.0), which defines having difficulty with an activity as increased effort, discomfort or pain, slowness or clumsiness, or changes in the way or manner in which the activity is performed [21]. Thus, we specifically focused on capturing “sensations” (pain, effort, slowness, compensation) associated with limitations, since, according to the ICF, they must be assessed to consider limitation of performance of any activity [10]. Additionally, interviews were conducted with gynecologists, members of an endometriosis patient association, and women awaiting consultations in hospital settings to capture diverse perspectives on how endometriosis influences disability, participation, and functioning. Whenever potential conceptual discrepancies could arise, they would be discussed and solved by a panel. Based on these findings, the research team generated an initial item pool comprising 21 items. The COSMIN guidelines for patient-reported outcome measures were followed as psychometric standards to strengthen the methodological framing of the study [22].

In summary, women identified seven activity domains that were most limited due to endometriosis: “bending down,” “rising from a seated position,” “standing up from a supine position,” “climbing stairs,” “straightening up,” “carrying heavy objects”, and “moving with heavy objects”. Based on the main sensations (i.e., effort, slowness, and/or pain) associated with limitations in these activities, three items were created for each domain (one for each sensation). All the aforementioned items included a question that was phrased as follows: “When you performed the activity X in the last 2 days, did your sensation of effort (or slowness or pain) increase in relation to the ideal perception of no sensation?”. The response format consisted of an 11-point Likert scale, where 0 indicated complete absence, 1 represented “very little,” and 10 corresponded to “the maximum imaginable”. For each activity, thus, difficulty was assessed from three perspectives: pain, effort, and slowness.

Following review by different stakeholders, the questionnaire was piloted with 20 patients with a confirmed medical diagnosis of endometriosis (who were not part of the final study sample), with the aim of ensuring clarity and comprehensibility. Scattered simple and minor formal modifications stemmed from the pilot. Face validity (the extent to which a measure looks like it will measure the thing it purports to) was then assessed and confirmed [23]. The sample was purposively selected to include adult women who were fluent in spoken and written Spanish. Efforts were made to include women with varying levels of pain, educational backgrounds, and ages. Data collection was conducted in person in the hospital waiting area, where members of the research team approached women with endometriosis and invited them to complete the questionnaire. A researcher remained nearby to discuss the relevance and clarity of the questions and to assist participants in expressing limitations in daily activities as accurately as possible.

### 2.3. Data Collection

The study was approved by the Clinical Research Ethics Committee of the Virgen de la Arrixaca University Hospital under the following code: 2023-2-5-HCUVA. Participants were informed of the confidential treatment of their data, as well as the voluntary nature of their participation in the study. All participants signed an informed consent form, and the guidelines of the Declaration of Helsinki were followed.

Patients were informed by the research team and the attending gynecologist about the voluntary nature of participation. Those study subjects meeting the inclusion criteria received detailed study information, reviewed the information sheet, signed the corresponding informed consent, and subsequently completed an initial questionnaire including sociodemographic (age, educational level, employment status) and the following clinical variables: height and weight (from which body mass index (BMI) was calculated), menstruation, pharmacological treatment for endometriosis, previous surgery for endometriosis, baseline pain assessment through the Numeric Rating Scale (NRS), and scores on the MOS SF-36 Physical Functioning Scale (PF-10) [24], the EHP questionnaire pain scale [7], and the MobEndo Questionnaire.

### 2.4. Data Analysis

A descriptive analysis of the sample’s sociodemographic variables was performed. Frequencies and percentages were calculated for categorical variables, while measures of central tendency and dispersion were obtained for quantitative variables.

Psychometric evaluation of the questionnaire was conducted with a sample of patients diagnosed with endometriosis at the Virgen de la Arrixaca University Hospital (Murcia, Spain). The initial sample was split into two equivalent subsamples following the “Solomon” method to conduct both an exploratory factor and a confirmatory factor analysis. The equivalence of both subsamples was tested through calculation of the communality ratio [25]. Thus, an exploratory factor analysis (EFA) [26] was carried out, using principal component analysis with Promax rotation. The specific choice of Promax rotation was reinforced a posteriori based on the excellent correlation (r = 0.821; *p*-value < 0.01) between both extracted components. Items with factor loadings higher than 0.40 on more than one factor were removed [27]. Structural validity was examined through Bartlett’s test of sphericity and the Kaiser–Meyer–Olkin (KMO) measure: values ≥ 0.60 were deemed acceptable. Confirmatory factor analysis (CFA), a structural equation modeling technique, was subsequently performed with the extant subsample to verify the factor structure. Factor loadings were calculated, aside from the factor-to-factor covariance. The general fit of the model was also assessed, by the comparative fit index (CFI), the Tucker-Lewis index (TLI), and the Root Mean Square Error of Approximation (RMSEA) [28].

The internal consistency of the structural components of the questionnaire was assessed using Cronbach’s alpha, considering values ≥ 0.70 as acceptable. To support the interpretation of internal consistency, inter-item correlation was also assessed, adopting the 0.3-to-0.9 acceptability values defined elsewhere [29]. To assess the test–retest reliability of the instrument, subjects completed the questionnaire twice on the same day, with an interval of approximately 30–45 min, to evaluate temporal stability [30]; for this purpose, a total of 30 individuals was selected, above the threshold of 15–20 participants that has been suggested as sufficient elsewhere [31]. Intraclass correlation coefficients were calculated with a two-way mixed-effect, single-rater, absolute agreement model ICC(3,2) and were judged as excellent if ICC > 0.75 [30].

Construct validity was evaluated through the assessment of concurrent, discriminant, and known-groups validity. Concurrent validity was evaluated by calculating Pearson or Spearman correlations (depending on data distribution) between the MobEndo questionnaire scores and both the PF-10 and the pain subscale of the EHP. Discriminant validity was assessed through correlations with variables theoretically unrelated to disability (age and BMI), where low or non-significant correlations were expected. On another note, known-groups validity was examined by defining three groups based on baseline pain levels (NRS): 0–3 indicated mild pain, 4–6 moderate pain, and 7–10 severe pain. This categorization is supported by recent clinical studies in endometriosis populations, where these thresholds have been used to stratify pain intensity for dysmenorrhea, chronic pelvic pain, and other endometriosis-associated symptoms [32] and is, furthermore, consistent with broader literature in the field of chronic pain. We hypothesized that the scores for all of the scales from MobEndo would be lower in participants with high levels of pain. One-way ANOVA (and, if needed, Tukey HSD post hoc analysis) was used to test for a difference in the mean scale scores across the aforementioned three pain-based groups.

The minimal sample size needed was set at n = 100, given that it corresponds to the minimal number determined for the examination of factor analysis and internal consistency, with a suggestion of 3 to 20 subjects per variable and absolute ranges from 100 to 1000 [33]. Additionally, the recommendations of Streiner et al. [34] were widely respected, suggesting a ratio of 5–10 participants per item for questionnaire validation.

Data analysis was conducted using IBM SPSS Statistics for Windows, version 28.0 (IBM Corp: Armonk, NY, USA). A confidence level of 95% was adopted, with the subsequent assumption of significance set at *p* < 0.05. More specifically, CFA was conducted with AMOS software version 26.0 (IBM Corp: Armonk, NY, USA) [35].

## 3. Results

### 3.1. Sociodemographic Characteristics of the Sample

Initially, a total of 326 potentially eligible subjects were invited to participate; amongst them, 20 declined participation (6.2% of the sample). Thus, 306 women engaged in the study. Five participants did not fully complete the MobEndo questionnaire due to missing responses on certain clinically related items. In total, therefore, 301 women successfully completed the questionnaire, and amongst them, a subsample of 30 women participated in the test–retest phase. The sociodemographic and clinical characteristics of the participants are displayed in Table 1.

### 3.2. Instrument Structure

The initial sample was split into two different subsamples (for the EFA and CFA, encompassing 150 and 151 subjects, respectively), with a communality ratio of 0.94, therefore confirming the equivalence of the corresponding subsamples [25].

During the initial inspection of the results from the preliminary exploratory factor analysis (EFA) that was performed, three items were identified for removal, since they exhibited factor loadings beyond 0.40 on more than one factor. Consequently, a set of 18 items was retained for the final analyses. To analyze the instrument structure, an EFA was performed. The Kaiser–Meyer–Olkin (KMO) index was 0.911, indicating excellent sampling adequacy. Bartlett’s test of sphericity was significant (χ^2^ = 3136.326; df = 153; *p* < 0.001), confirming the suitability of the data for factor analysis. The EFA revealed a two-component structure with eigenvalues greater than 1, explaining 71.78% of the total variance. Component 1 comprised 12 items, and component 2 included 6 items. Based on their item-based content, the components were labeled as “transitioning between body positions and performing movements requiring stabilization” (items 1 to 12) and “executing load-bearing tasks involving the upper limbs” (items 13–18), with adequate factor loadings across all items. Further data on the EFA that was performed are available in Table 2.

**Figure 1:**
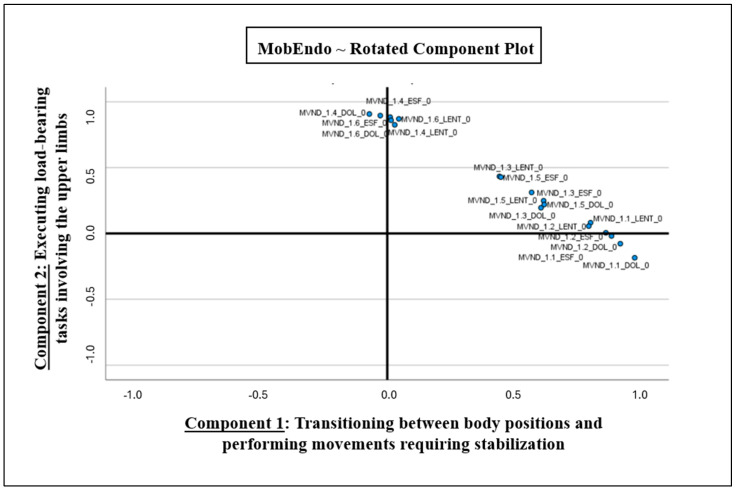
Rotated component plot. Distribution of questionnaire items across two components after Promax rotation. Each point represents an item, positioned according to its factor loadings on component 1 (horizontal axis) and component 2 (vertical axis). Note: MVND_1.n_DOL_0 corresponds to the “n” item of MobEndo focusing on pain, whilst the suffix LENT corresponds to slowness, and ESF to effort.

The rotated component plot (Figure 1) illustrates the distribution of questionnaire items across two distinct components identified through the EFA. Component 1 primarily captures limitations related to transitions between body positions and movements requiring pelvic and abdominal stabilization, such as bending down, rising from a seated position, and standing up from a supine position, including climbing stairs, whilst component 2 reflects difficulties associated with trunk extension and load-bearing tasks involving the upper limbs, straightening up, and carrying heavy objects. The clear separation of items across these components supports the structural validity of the instrument and confirms that the questionnaire effectively distinguishes between different functional domains impacted by endometriosis-related disability.

**Figure 2:**
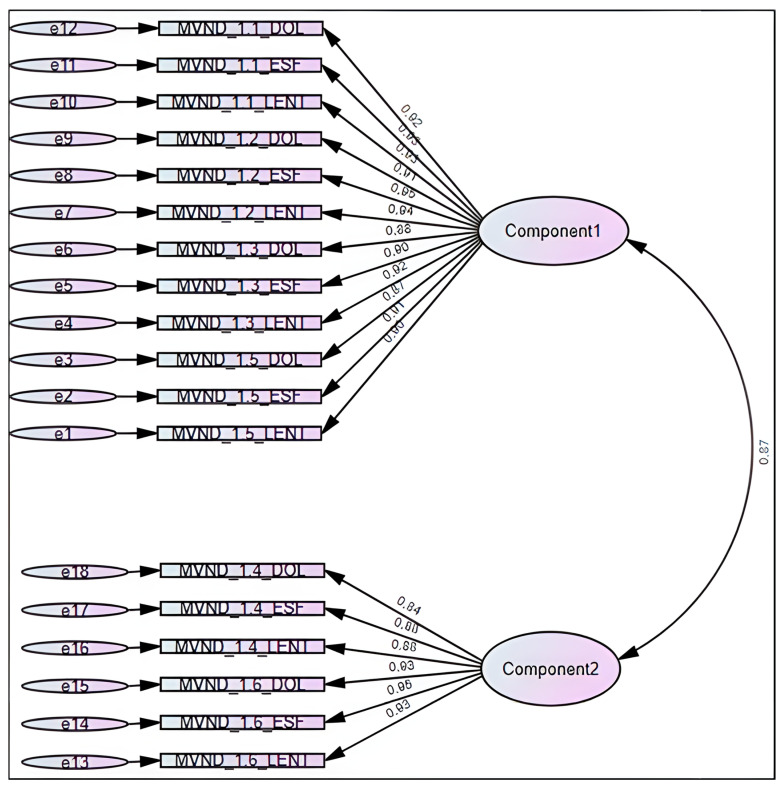
Structural equation modeling for confirmatory factor analysis of the MobEndo instrument. Note: MVND_1.n_DOL_0 corresponds to the “n” item of MobEndo focusing on pain, whilst the suffix LENT corresponds to slowness, and ESF to effort.

The structural equation modelling performed for CFA yielded factor loadings in a 0.87-to-0.95 and 0.84-to-0.95 range for components 1 and 2, respectively, as shown in Figure 2. As for the model fit statistics (χ^2^ = 1463.499; df = 134: *p* < 0.001), the CFI corresponded to 0.746, whilst the TLI was 0.710, and the RMSEA corresponded to 0.256.

### 3.3. Reliability

The questionnaire demonstrated excellent internal consistency, with an overall Cronbach’s alpha of 0.977. The Cronbach’s alpha values for the components ranged from 0.952 to 0.972, indicating strong homogeneity among items. The average inter-item correlations corresponded to 0.743 (range: 0.631–0.907) for component 1, 0.775 (range: 0.664–0.905) for component 2, and 0.699 (range: 0.539–0.907) for the overall instrument. As for the test–retest reliability of the instrument, the ICC obtained for the whole instrument was 0.976 (95% CI 0.949–0.988), while those for components 1 and 2 were 0.977 (95% CI 0.951–0.989) and 0.964 (95% CI 0.925–0.983), respectively, reflecting excellent stability across time. Further information is provided in Table 3.

### 3.4. Construct Validity

Significant correlations were observed between the total score for the MobEndo questionnaire and its two components and both the PF-10 and the pain scale of the EHP, therefore displaying good concurrent validity. In contrast, correlations between the questionnaire and variables theoretically unrelated to disability, such as age and BMI, were low and non-significant, confirming the instrument’s discriminant validity. Focusing on the known-groups validity, the hypothesis was tested, and the three performed one-way ANOVA analyses yielded *p*-values < 0.001 (with F-scores of 168.04, 129.75, and 181.43 for component 1, component 2, and the global instrument, respectively). The post hoc (Tukey HSD) analyses performed displayed statistically significant differences among the three pain-based groups across the two components and the overall tool. Specific data on the different forms of construct validity conducted are displayed in Table 4.

## 4. Discussion

The objective of the current study was to develop and analyze the psychometric properties of a new instrument to identify and quantify the functional difficulties faced by women with endometriosis. The questionnaire was developed based on the theoretical framework of the ICF [10]. Women with endometriosis not only report pain, but they also describe difficulties in performing daily activities. According to the ICF principles, when an activity is performed with more pain, greater effort, more slowly, or in a different manner, this reflects a performance or capacity problem within the Activities and Participation components. Such difficulties may arise from increased time required, fatigue or pain, need for assistance, or compensatory movement patterns. All these principles guided the underlying philosophy for the creation and development of the questionnaire. By offering a structured, patient-centered approach to functional assessment, the proposed questionnaire has the potential not only to enhance clinical understanding, but moreover to guide therapeutic interventions and support more comprehensive care strategies.

The results stemming from the initial psychometric testing demonstrated adequate structural configuration of the instruments, indicating that the items accurately and clearly reflect the intended construct and domain [36], supported by the KMO test and Bartlett’s sphericity test. Exploratory factor analysis identified two main components: (1) transitioning between body positions and performing movements requiring stabilization (items 1 to 12) and (2) executing load-bearing tasks involving the upper limbs. The resulting components and the item aggregation around them are consistent with the theoretical principles of musculoskeletal function, and they also fall in line with the current scientific literature, since previous studies have shown that women with endometriosis often exhibit reduced flexibility and functional impairments in the lumbopelvic region, as well as kinesiophobia and disability related to movement tasks [37]. Interventions targeting lumbopelvic stabilization and strengthening have demonstrated improvements in pain and physical function, reinforcing the relevance of these components for clinical assessment [38]. Furthermore, questionnaires such as the EIQ are conceptually designed as measures of impact on health-related quality of life, while still allowing the extraction of a physical or functional dimension score. This aspect is reflected in items addressing pain, fatigue, limitations in daily activities, and the extent to which endometriosis restricts participation in usual roles over time. The inclusion of physical domains related to posture and mobility, as in the MobEndo instrument, further reinforces the conceptual validity of these components [8]. These findings highlight the importance of distinguishing between difficulties related to pelvic-abdominal control and those involving trunk extension and load-bearing activities.

The confirmatory factor analysis conducted yielded excellent results in terms of factor loading, since almost all the items were loaded above and beyond 0.90. However, two specific drawbacks arouse: the factor-to-factor covariance, indicating how latent factors “move” together, was high, suggesting high collinearity and thus a potential issue in terms of discriminant validity between them. Also, the model fit statistics performed poorly, since neither the CFI nor the TLI reached the 0.90 threshold for acceptable fit, and the absolute fit index, the RMSEA, assessing how far a hypothesized model is from a perfect model, was poor. The combination of low CFI and TLI with high RMSEA values may stem from a relatively small sample, a fact that shall be taken into account when conducting further psychometric testing of the instrument [39].

The psychometric evaluation performed on the new instrument also revealed satisfactory reliability. These results concerning MobEndo fall along the lines of both the EIQ [8] or the EHP-30 [7], therefore reinforcing and highlighting the instrument’s consistency in measuring limitations to the performance of mobility activities.

The results stemming from our study shall nonetheless be interpreted in the light of its methodological limitations. First, external validity could not be assessed due to the absence of standardized instruments measuring the same constructs. Second, the instrument was solely cross-sectionally tested; further studies should focus on its performance over time, to capture the sensitivity to change of the instrument. Third, the interval adopted for the test–retest reliability measure of the questionnaire corresponded to a 30-to-45 min window: while 30 min is sufficient for physical recovery, our instrument involved cognition and, thus, this interval may not completely eliminate the effects of memory or short-term mood fluctuations, therefore introducing potential bias. Fourth, some specific properties (e.g., measurement error, interpretability) were not assessed, and they should be evaluated in further psychometric testing and validations. Fifth, the sample primarily comprised women with moderate-to-high education levels (61.2% university-educated), which may limit generalizability to populations with lower educational attainment. Finally, the sample consisted exclusively of patients from a single hospital (single-center design) and from a specific hospital-based setting, limiting the generalizability of the findings. Future research should focus on cross-cultural adaptation and validation of the questionnaire in different countries to enable comparative analyses and to deepen understanding of the phenomenon. Additional studies are needed to confirm the validity and reliability of this instrument across diverse endometriosis populations.

The development of the current instrument opens a new window into the clinician–patient relationship, since it provides the former with a deeper understanding of the functional limitations of their patients and, consequently, emerges as a fundamental evaluative tool in the progress towards a strategy of individual patient management in clinical practice, identified as one of the main challenges in rehabilitation care [40].

The practical implications of assessing disability from a biopsychosocial perspective, and grounded in the ICF framework, may provide a more accurate and holistic understanding of how endometriosis affects movement, which, in turn, and in conjunction with other assessments (pain scales, quality-of-life measures) may help clinicians to develop comprehensive treatment plans. The development and validation of this specific instrument to evaluate functional disability in this population is important for improving clinical assessment, monitoring therapeutic progress, and designing personalized and tailored physiotherapy interventions.

## 5. Conclusions

This study evinces that the questionnaire developed has demonstrated validity and reliability in a specific sample and context for capturing the perceptions of women with endometriosis of their mobility limitations. Psychometric analysis revealed two underlying components: (1) transitioning between body positions and performing movements requiring stabilization and (2) executing load-bearing tasks involving the upper limbs. Further studies shall focus on validation of the instrument in different contexts, as well as in broader and more heterogeneous sample populations.

## Figures and Tables

**Table 1:** Characteristics of participants from the sample and subsample for test–retest.

Variable		n	Mean ± SD N (%)	N	Mean ± SD N (%)
Age		301	38.96 ± 6.85	30	36.33 ± 7.40
	Up to 39 years		138 (45.8)		15 (50.0)
	40 years or older		163 (54.2)		15 (50.0)
Education level		273		29	
	Elementary		14 (5.1)		0 (0.0)
	Secondary		92 (33.7)		6 (20.7)
	University		167 (61.2)		23 (79.3)
Current professional status		298		30	
	Housewife		16 (5.4)		0 (0.0)
	Currently working		229 (76.8)		25 (83.3)
	On medical leave		13 (4.4)		3 (10.0)
	Unemployed		37 (12.4)		2 (6.7)
	Pensioner		3 (1.0)		0 (0.0)
Body mass index (BMI)		281		28	
	<25		174 (61.9)		19 (67.9)
	≥25		107 (38.1)		9 (32.1)
Presence of menstrual cycle (Yes)		297	187 (66.3)	30	20 (66.7)
NRS baseline pain		301		30	
	None to mild (0–3)		55 (18.3)		8 (26.6)
	Moderate (4–6)		163 (54.1)		17 (56.7)
	Severe (7–10)		83 (27.6)		5 (16.7)
Pharmacological treatment for endometriosis? (Yes)		300	210 (70.0)	30	26 (86.7)
Surgery for endometriosis (Yes)		301	132 (43.9)	30	6 (20.0)
EHP-Pain		297	33.26 ± 26.79	30	27.50 ± 14.77
PF-10 (percentage: 0–100)		300	74.01 ± 24.85	30	74.35 ± 21.12

SD: Standard deviation; BMI: body mass index; NRS: Numeric Rating Scale; EHP-Pain: pain scale of the EHP questionnaire; PF-10: MOS SF-36 Physical Functioning Scale.

**Table 2:** Factor loadings for each item and component assignment from the exploratory factor analysis (n = 150).

Items	Component 1	Component 2
Getting up after sitting for a while is more painful than when you are perfectly well.	0.977	
Getting up after sitting for a while requires more effort than when you are perfectly well.	0.920	
Standing up after bending down to pick up an object from the floor (or washing machine/dishwasher) is more painful than when you are perfectly well.	0.885	
Standing up after bending down to pick up an object from the floor (or washing machine/dishwasher) requires more effort than when you are perfectly well.	0.863	
When getting up after sitting for a while, you do it more slowly than when you are perfectly well.	0.802	
When standing up after bending down to pick up an object from the floor (or washing machine/dishwasher), you do it more slowly than when you are perfectly well.	0.795	
Climbing stairs, is more painful than when you are perfectly well.	0.620	
Getting up from lying down in bed (or on a sofa) requires more effort than when you are perfectly well.	0.617	
Getting up from lying down in bed (or on a sofa) is more painful than when you are perfectly well.	0.607	
When climbing stairs, you do it more slowly than when you are perfectly well.	0.570	
Climbing stairs requires more effort than when you are perfectly well.	0.525	
When getting up from lying down in bed (or on a sofa), you do it more slowly than when you are perfectly well.	0.512	
Reaching up to place a small object on a high shelf is more painful than when you are perfectly well.		0.907
Carrying a heavy object inside the house (e.g., basin, bag) by holding it with arms and hands (as if carrying a baby) requires more effort than when you are perfectly well.		0.895
Reaching up to place a small object on a high shelf requires more effort than when you are perfectly well.		0.883
When carrying a heavy object inside the house (e.g., basin, bag) by holding it with arms and hands (as if carrying a baby), you do it more slowly than when you are perfectly well.		0.870
Carrying a heavy object inside the house (e.g., basin, bag) by holding it with arms and hands (as if carrying a baby) is more painful than when you are perfectly well.		0.860
When reaching up to place a small object on a high shelf, you do it more slowly than when you are perfectly well.		0.825

Exploratory factor analysis: principal component analysis with Promax rotation.

**Table 3:** Internal consistency and test–retest reliability.

Component	Cronbach’s Alpha	ICC (95% CI)
Overall	0.977	0.976 (0.949–0.988)
Component 1—Transitioning between body positions and performing movements requiring stabilization	0.972	0.977 (0.951–0.989)
Component 2—Executing load-bearing tasks involving the upper limbs	0.952	0.964 (0.925–0.983)

**Table 4:** Construct validity (n = 301).

Form of Construct Validity	Concurrent		Discriminant		Known-Groups		
Analysis	Pearsons’s Correlation Coefficient, *p*-Value		Pearsons’s Correlation Coefficient, *p*-Value		ANOVA Eta-Squared, F (df), *p*-Value		
Variable	PF-10 (n = 300)	EHP Pain (n = 301)	Age (n = 299)	BMI (n = 281)	None-Mild (n = 55)	Moderate (n = 163)	Severe (n = 83)
**Component 1**	−0.717; <0.001	0.651; <0.001	0.009; 0.874	0.109; 0.068	0.470, 168.04 (2); 0.001 *		
**Component 2**	−0.640; <0.001	0.609; <0.001	−0.045; 0.442	0.061; 0.311	0.448, 129.57 (2); 0.001 *		
**Overall**	−0.722; <0.001	0.666; <0.001	−0.010; 0.866	0.098; 0.102	0.501, 181.43 (2); 0.001 *		

Df: degrees of freedom; PF-10: MOS SF-36 Physical Functioning Scale; BMI: body mass index; EHP Pain: pain scale of the EHP questionnaire. * Tukey post hoc analysis stating statistically significant (*p* < 0.001) differences across the three groups (none-mild, moderate, and severe).

## Data Availability

The data that support the findings of this study are available on reasonable request from the corresponding author.

## Abbreviations

The following abbreviations are used in this manuscript:

BMI	Body mass index
CFA	Confirmatory factor analysis
CFI	Comparative fit index
Df	Degrees of freedom
EFA	Exploratory factor analysis
EHP	Endometriosis Health Profile
EIQ	Endometriosis Impact Questionnaire
HSD	Tukey Honest Significant Difference
ICC	Intraclass correlation coefficients
ICF	International Classification of Functioning, Disability, and Health
KMO	Kaiser–Meyer–Olkin test
NRS	Numeric Rating Scale
PF-10	MOS SF-36 Physical Functioning Scale
PROMs	Patient-reported outcome measures
RMSEA	Root mean square error of approximation
SD	Standard deviation
TLI	Tucker–Lewis index
